# Classical and Innovative Evidence for Therapeutic Strategies in Retinal Dysfunctions

**DOI:** 10.3390/ijms25042124

**Published:** 2024-02-09

**Authors:** Lorenzo Caruso, Matteo Fields, Erika Rimondi, Giorgio Zauli, Giovanna Longo, Annalisa Marcuzzi, Maurizio Previati, Arianna Gonelli, Enrico Zauli, Daniela Milani

**Affiliations:** 1 Department of Environmental and Prevention Sciences, University of Ferrara, 44121 Ferrara, Italy; lorenzo.caruso@unife.it (L.C.); arianna.gonelli@unife.it (A.G.); 2 Department of Translational Medicine, University of Ferrara, 44121 Ferrara, Italy; matteo.fields@unife.it (M.F.); giovanna.longo@unife.it (G.L.); annalisa.marcuzzi@unife.it (A.M.); maurizio.previati@unife.it (M.P.); daniela.milani@unife.it (D.M.); 3 Department of Translational Medicine and LTTA Centre, University of Ferrara, 44121 Ferrara, Italy; 4 Research Department, King Khaled Eye Specialist Hospital, Riyadh 11462, Saudi Arabia; giorgio.zauli@unife.it

**Keywords:** retinopathies, inflammation, gut–retina axis, retinal organoid

## Abstract

The human retina is a complex anatomical structure that has no regenerative capacity. The pathogenesis of most retinopathies can be attributed to inflammation, with the activation of the inflammasome protein platform, and to the impact of oxidative stress on the regulation of apoptosis and autophagy/mitophagy in retinal cells. In recent years, new therapeutic approaches to treat retinopathies have been investigated. Experimental data suggest that the secretome of mesenchymal cells could reduce oxidative stress, autophagy, and the apoptosis of retinal cells, and in turn, the secretome of the latter could induce changes in mesenchymal cells. Other studies have evidenced that noncoding (nc)RNAs might be new targets for retinopathy treatment and novel disease biomarkers since a correlation has been found between ncRNA levels and retinopathies. A new field to explore is the interaction observed between the ocular and intestinal microbiota; indeed, recent findings have shown that the alteration of gut microbiota seems to be linked to ocular diseases, suggesting a gut–eye axis. To explore new therapeutical strategies for retinopathies, it is important to use proper models that can mimic the complexity of the retina. In this context, retinal organoids represent a good model for the study of the pathophysiology of the retina.

## 1. Functional Anatomy of Retina: Morphofunctional Characterization

Among the three layers of the eye, the retina represents the innermost, located just between the vitreous body and the choroid. In the retina, ten different layers can be observed, composed of six different types of neuronal cells, involved in the creation and transmission of the visual signal. These layers are arranged in a precise order from the inner layer (anterior) to the outer layer (posterior): 1. Inner limiting membrane; 2. Nerve fiber layer; 3. Ganglion cell layer; 4. Inner plexiform layer; 5. Inner nuclear layer (INL); 6. Middle limiting membrane; 7. Outer plexiform layer; 8. Outer nuclear layer (ONL); 9. External limiting membrane; 10. Photoreceptor outer segments. The cells that constitute these layers are rods, cones, retinal ganglion cells (RGCs), bipolar cells, horizontal cells, and amacrine cells. All these cell types have a specific role and constitute the circuitry that allows the retina to detect and signal every change of the light [1]. The inner retina receives oxygen and nutrient supply from the microvasculature that forms the inner blood–retina barrier (BRB). This term refers to the complex architecture of the blood vessels that irrorate the nerve fiber layer, the inner plexiform layer, and the outer plexiform layer. These vessels consist of endothelial cells presenting tight junctions that avoid any leakage, surrounded by pericytes and glial cells that support them, form a more intact barrier against the diffusion of molecules from the bloodstream, and regulate proliferation and angiogenesis. These cells constitute the so-called neurovascular unit, that resembles the blood–brain barrier in its structure and functions [2]. The complex structure of the retina is externally lined by Bruch’s membrane, which separates the fenestrated capillaries of the choroid from the retinal pigment epithelium (RPE). Bruch’s membrane, being formed by elastic and collagen fibers, holds a structural role, contributing to counteract internal ocular pressure (IOP), to limit cell migration from external layers, and control nutrient diffusion from the choroid fenestrated capillaries on the basis of molecular weight. Together, Bruch’s membrane, the choroid capillaries, and their basal lamina constitute the outer BRB. Internally to Bruch’s membrane, there is the RPE layer, immediately followed by three main neuron cell types, arranged in the following order: photoreceptors, bipolar cells, and ganglion cells, associated with other cells modulating neurotransmission [3,4,5]. Photoreceptors are specialized neurons that extend between the ONL and the photoreceptor outer segments, and are divided in two subtypes: rods (95%) and cones (5%). The largest rod concentrations are seen in the outer retina, with density increasing toward the retina’s periphery. The axons of several rods form synapses with a single RGC, providing information about peripheral and scotopic visions during nighttime; as we move towards the peripheral areas, they reach a ratio of a million rods per single RGC. While rods are more receptive to individual photons of light, cones are less sensitive to photons in general, but they respond to one of three specific wavelengths of light (red, green, and blue colors). Because this information has a heavier weight in image-forming processes in the brain, there is a lower ratio of synapses between cones and RGCs, as low as 1:1 in the macula region of the retina [6]. The primary output neurons in the retina are RGCs. These cells also function as a third class of photoreceptors that plays an important role in the transmission of both image-forming and non-image-forming information [7]. Different inputs are transmitted to RGCs by two types of intermediate neurons, bipolar cells and amacrine cells. Bipolar cells are secondary neurons, characterized by two long projections that allow them to relay the inputs from photoreceptors to RGCs. They lie in the INL, with a synaptic connection in the OPL and the other in the INL. They also interact with amacrine cells and horizontal cells, and thus they are essential for processing the inputs from photoreceptors via the modulation of neuronal transmission mediated by these interneurons [8]. Amacrine cells have the role of intermediate neurons in the retinal circuit, providing inhibitory GABAergic signals. Several amacrine cells contact the same RGC, and a single amacrine cell can contact different RGCs, forming a microcircuit responsible for transmitting information about different shades and movements of light [9]. The last type of neuronal cells is represented by horizontal cells, that act as modulators of the signal transfer from photoreceptors to bipolar cells, particularly important for the eyes to adapt to various conditions of light intensity. They lie in the INL with their projections contacting photoreceptors’ synapses in the OPL [10]. In addition to these neuronal cell types, the retina also comprises several types of glial cells, represented by Müller cells, astrocytes, and microglia. In particular, Müller cells constitute a large part of the volume of the retina and extend from the inner limiting membrane, which they contribute to form with their basal expansion, to the ONL. Müller cells are placed in the space among neurons, isolating them from each other, except for the synaptic contacts. They are in a sort of symbiotic relationship with neurons, being involved in the proper functioning of synapses, the neurodevelopment of the retina, neuronal plasticity, and protection from mechanical stress. Müller cells also exert control over the extracellular environment to prevent harmful changes for the neurons: they regulate the extracellular concentration of neuroactive substances (potassium, GABA, and glutamate neurotransmitters) as well as ions, bicarbonate, and water. They may also contribute to the processing of the visual information in the retina, by producing, storing, and releasing neuroactive substances in response to neuronal activation [1,11].

**Figure 1:**
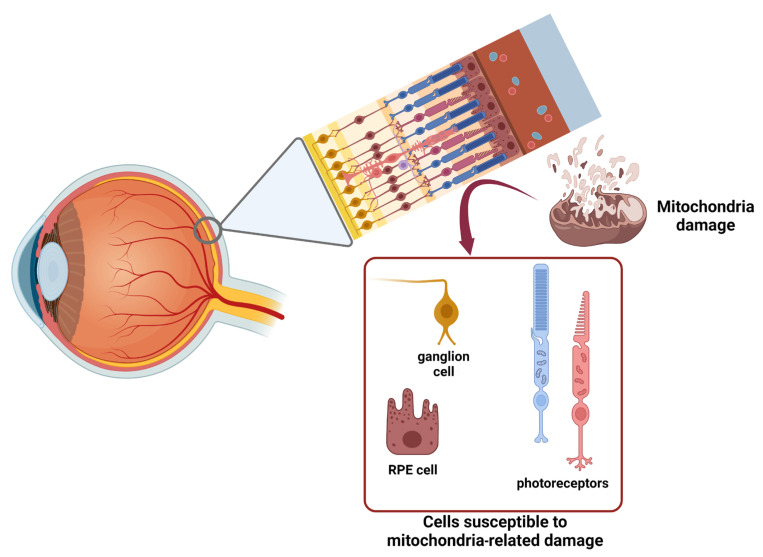
Mitochondria-related damage on RPE, photoreceptors, and ganglion cells in retinal disease. Created with BioRender.com.

The RPE and photoreceptors (rod and cones) constitute the outer part of the retina, that is avascular and depends for oxygen and nutrient supply, as well as waste removal, on the choroid capillaries. Interestingly, photoreceptors account for 110–130 million of cells, and are a highly metabolically active cell type that consumes roughly 75% of the available oxygen. On the other hand, the outer part of the retina is quite a hypoxic environment, which prompts RPE cells to assume several different supporting functions. RPE cells, which intercalate with their microvilli among photoreceptors, furnish a wide exchange surface that phagocytizes the outer parts of photoreceptors, shuttles glucose and oxygen, and recycles chromophores for visual pigments. Moreover, RPE cells are responsible for phagocytosis and recycling of photoreceptor outer segments (POS) fragments, shed after they accumulated toxic compounds. This phagocytic activity reaches its peak 1–2 h after light exposure at the beginning of the day, pointing to a possible contribution of the circadian clock in this process. About this topic there are controversial results. Some researchers observed that the disruption of the circadian rhythm was responsible for the accelerated degeneration of the photoreceptor layer due to impaired phagocytosis by the RPE, leading to the accumulation of ROS and undegraded waste products [12]; others instead showed that, in vivo, the disruption of the phagocytosis peak dependent on the circadian rhythm was not associated with photoreceptor degeneration, because the RPE could activate a compensatory mechanism that increased baseline phagocytic activity [13,14]. In addition, in the semi-hypoxic environment of the outer retina, the photoreceptors’ viability relies not only on mitochondrial oxidative metabolism, but also on cytoplasmic glycolysis, producing large amounts of lactate, which is shuttled to RPE and Müller cells to be oxidized to pyruvate. In this view, the availability of RPE and Müller cells to use pyruvate, saving and furnishing glucose for photoreceptor metabolism, is of primary importance [15]. On the other hand, fatty acid oxidative metabolism has been reported to contribute to fulfilling the bioenergetic needs of neuronal metabolism [16]. In addition to photoreceptors and the RPE, RGCs represent another subset of retinal cells that display a particular susceptibility to mitochondria-related damage, in this case, by virtue of their particular disposition in the tissue architecture (Figure 1) [17]. The bodies of RGCs, in fact, develop axonal segments that run over the inner aspects of the retina, are interposed on the light path, and consequently must be unmyelinated for the whole length of the retinal route, becoming able to acquire a myelin sheath only after the lamina cribrosa, just inside the optic nerve. This leads to an asymmetric distribution of mitochondria in the different parts of the optic nerve, with a strong mitochondrial enrichment in the unmyelinated portion of the axonal segment. This asymmetric distribution can make the proximal part of the optic nerve more sensitive to mitochondrial malfunctioning, and consequently strictly dependent on the efficiency of quality control mechanisms of mitochondrial performance, such as autophagy, mitophagy, and mitochondrial biogenesis.

Alterations of different portions of the retina cause effects of different severity depending on the affected areas. Lesions affecting the central area of the retina (macula) can cause the loss of acuity of central vision, the distortion of straight lines, and the alteration of color vision. Lesions affecting parts of the retina other than the macula can cause changes in portions of the visual field or, in severe cases, lead to total blindness. The retina, in general, can be affected by different types of vascular or degenerative diseases resulting from other pathologies such as atherosclerosis, arterial hypertension, or diabetes.

## 2. Autophagy and Mitophagy: Programmed Cell ‘Amputations’ to Limit the Damage

The term autophagy identifies some lysosome-centered cellular pathways strongly conserved during evolution, devised to maintain cellular homeostasis by removing damaged material, supplying nutrients during metabolic stress, and trying to prevent genomic damage [18]. In particular, autophagic pathways identify, engulf, and carry to the lysosomal compartment a wide group of intracellular components, from low-size macromolecules to entire organelles. Autophagy is frequently described as relying on three different mechanisms, termed macroautophagy or more commonly autophagy, microautophagy, and chaperone-mediated autophagy. Macroautophagy is able to create an intracellular double membrane system to engulf portions of organelles or cytoplasm, with the formation of a vesicle called autophagosome. These vesicles are carried and fused to a lysosome, to allow for the degradation of the selected material. On the contrary, in microautophagy, the autophagosome is absent, and small portions of cytoplasm are incorporated into lysosomes through the invagination of the lysosomal membrane. In chaperone-mediated autophagy, the hsc-70 protein recognizes and binds a pentapeptide sequence of the cytoplasmic proteins to drive them to lysosomal degradation [19]. In addition to these widely studied and well-known examples of degradative pathways, there are also non-degradative pathways, where the autophagic proteins are also involved in membrane trafficking devoted to the exocytosis of secretory granules, interleukins, High Mobility Group Box 1 (HMGB1), exosomes, and others [20].

As a whole, the physiological value of autophagy lies in two main activities.

Autophagy acts as a quality control mechanism, able to reshape the cell through the removal of damaged proteins and organelles. Different forms of autophagy have been identified, based on what organelle/molecule needs to be degraded: mitochondria (mitophagy), endoplasmic reticulum (reticulophagy), peroxisomes (pexophagy), or lipid droplets (lipophagy). In addition, the removal of invading pathogens (xenophagy) warrants a role in cell defense [21]. The degradative aspect of autophagy constitutes a very important tool for the cell, because it warrants a vital source of amino acids and lipids for the de novo synthesis of proteins and lipids during periods of shortage. Overall, this concerns protein synthesis, which can be performed only in the contemporary presence of all the building blocks, in particular, essential amino acids. Under starvation or in the presence of stress conditions, amino acid pool completeness can be ensured only through the demolition of existing cellular proteins. Therefore, autophagy has a fundamental role in cell survival, because it not only provides the removal of dangerous cellular components, but also secures biosynthetic compounds [22]. So, by virtue of its basic function in cellular physiology, autophagy is involved in most human diseases, like cancer or neurodegeneration, where it can behave, according to the specific circumstances, as a double-edged sword, helping in counteracting the disease or fostering it.

A subset of autophagy-related molecular mediators are also involved in a non-canonical autophagy pathway, named microtubule-associated proteins light chain 3 (LC3)-associated phagocytosis (LAP) [23,24], in which single-membrane vesicles are formed. LAP is involved in immune-suppressive and anti-inflammatory responses, and its deficiency induces hyper-inflammation and cytokine production [25]. It has been shown that LAP has a fundamental role in maintaining the functions of different cell types, among which RPE [26]. In RPE cells, a homeostatic balance between autophagy, that plays an important role against oxidative stress [27,28], and LAP, that is involved in the degradation of POS fragments [29] and the synthesis of anti-inflammatory lipids [29], is maintained.

The control and reshaping of damaged mitochondrial population involve different mechanisms. A first mechanism is the continuous elimination of damaged proteins, operated by mitochondrial proteasome, mitochondrial unfolded protein response, or proteasome-dependent degradation [30]. In the presence of greater amounts of oxidized or damaged proteins and lipids, these can be packed into portions of the mitochondrion, that can subsequently be released as vesicles of 70–150 nm in diameter, called mitochondrial-derived vesicles (MDVs). Their fate is to be transported intracellularly to lysosomes and peroxisomes and degraded [31]. Mitophagy, as mentioned above, is a selective form of autophagy that destroys the whole mitochondrion. The fragmentation of the mitochondrion facilitates the overall mitophagy process, and potentially regulates it. In addition, mitochondria need to be marked on the surface by an “eat me” signal, to start the destructive route. One of the most studied signals is the ubiquitin-dependent PINK1/Parkin axis. PINK1 and Parkin are members of the family of PARK genes, which include α-synuclein (α-syn, PARK1/4), Parkin (PARK2), PINK1 (PTEN-Induced Kinase 1-PARK6), DJ-1 (PARK7), LRRK2 (PARK8), and ATP13A2 (PARK9). The finding that mutations in these proteins are related to inherited forms of Parkinson’s disease not only gave this group of genes its name, but also highlights the tight link between mitophagy regulation and neuropathies [32]. The PINK1/Parkin axis regulates mitophagy firstly through the degradative cycle of PINK1. PINK1 is an ubiquitous serine/threonine-protein kinase imported into mitochondria by the action of the protein translocators TIM and TOM. Once arrived in the inner mitochondrial membrane, PINK1 under normal conditions is proteolytically degraded [33,34,35]. Instead, in the presence of alterations of mitochondrial membrane potential, PINK1 accumulates on the external mitochondrial membrane where it is stabilized by a molecular complex including TOM proteins [36,37] and begins to phosphorylate Parkin. This post-translational modification allows Parkin to become an active Ub-dependent enzyme [38,39] which ubiquitinates several mitochondrial proteins of the external membrane. Several proteins, like p62/Sequestosome, NBR1, NDP52, optineurin (OPTN), and TAX1BP1 (TBK1), recognize the ubiquitinated proteins and mediate the docking of the damaged mitochondria to the autophagosomes for clearance in the lysosome [40,41,42].

## 3. Etiopathogenesis and Characterization of Retinopathies: Two Meaningful Examples

Oxidative stress is regarded as one of the triggers that cause cellular dysfunction and tissue damage in many retinal diseases. Oxidative stress is caused by ROS generation and accumulation, mostly sourced from mitochondria during oxidative phosphorylation. Therefore, mitochondria need an efficient antioxidant system to counter ROS accumulation. As explained in the previous section, when this mechanism fails, ROS may attack proteins and mtDNA, forcing the cell to get rid of the damaged portion of mitochondria through mitophagy.

There is a connection between oxidative stress and inflammation: dying cells that experienced oxidative stress may release factors that induce TNF-α production in macrophages and healthy RPE cells. At the same time, proinflammatory cytokines like TNF-α, IL-1β, and IFN-γ are able to increase ROS production in RPE cells, as observed in patients with macular degeneration and diabetic retinopathy (DR) [43]. This further proves that inflammation is another key element in the insurgence of retinopathies and maculopathies [44,45]. The catalyst of this process is the activation of the inflammasome protein platform and in particular pyrin domain-containing protein 3 (NLRP3). The activation of the inflammasome and the subsequent release of caspases are the first steps of the inflammatory response due to the conversion of some interleukins, in particular, pro-IL-1b and pro-IL-18, to their active forms. Further evidence has shown that the inhibition of the NLRP3-inflammasome can lead to a decrease in pro-inflammatory molecules, and a consequent decrease in vascular permeability [46,47]. It is necessary to consider that vascular phenomena play an important role in acute inflammatory response. Indeed, the blood vessels of the microcirculation undergo modifications that represent a prerequisite for the leakage of the main effectors of the defense mechanisms at the site of injury or infection.

Regardless of the cause (e.g., age, hypoxia, hyperglycemia, or genetic factors), there is a reduction in the effectiveness of the disposal of cellular debris by macrophages, and this is particularly evident in the RPE. Under physiological conditions, microglia cells migrate to the site of damage, engulf the apoptotic material, and activate the complement system, to properly manage the cellular debris produced, and the assembly of the inflammasome. When this mechanism escapes normal control, there is a real danger to the surrounding tissue, because if the insult persists, it can cause tissue remodeling [48,49,50].

Various proinflammatory factors, including reactive oxygen species (ROS), TNF-α, and complement activators, are released by overly activated microglia and act as enhancing triggers for inflammasome assembly. This generates a chronic inflammatory response, that alters the function and structure of the RPE, the permeability of the BRB, the formation of new vessels, and the recruitment of macrophages at the choroid level [51]. A crucial point of this mechanism is the role of neo-vascularization induced by the increased expression of vascular endothelial growth factor (VEGF) that can either be considered the cause of the activation of the inflammation itself or, according to other evidence, it could be its consequence [52,53,54].

### 3.1. Glaucoma

The term glaucoma indicates a heterogeneous group of maculopathies with an etiopathogenesis that is not fully clarified, which represents, worldwide, the second cause of blindness. Glaucoma is expected to affect more than 110 million of patients in 2040 and, if not treated, could inevitably lead to the degeneration of RGCs and the optic nerve, and consequently to vision loss. There are several known risk factors, including optic disc hemorrhage, systemic hypertension, diabetes mellitus, smoking, severe myopia, and lipid dysregulation, but IOP is one of the most relevant ones. In fact, disease progression is frequently related to IOP and can stop when pressure is reduced: consequently, glaucoma therapies overall rely on the management of IOP, both with pharmacological and surgical strategies [55]. A potential failure of IOP management opens the possibility of an irreversible degeneration of RGCs and their unmyelinated axons in the retinal nerve layer, followed by optic nerve depletion. A more precise evaluation of the molecular mechanisms actually involved in cellular degeneration is needed to develop innovative therapies to prevent the worst impact of the illness [56]. It should be noted that the implications of RGCs degeneration are not limited to the optic nerve but can spread and also involve other components of the visual pathway, like lateral geniculate nuclei and the visual cortex [56].

IOP has been involved in the reduction of blood flow in the choroid or in the vessels of the innermost part of the retina. This can produce retinal ischemia which appears to be common to several other retinopathies, including DR, and has been proposed as a reasonable cause of RGC injuries. In addition to vessel and tissue compression, a consequence of increased IOP is the stretching and thinning of all retinal structures. This occurrence, common to high myopia, can lead to stress in all the tissues composing the three layers of the eye. As a whole, oxidative stress, neuroinflammation, and ischemia are surely involved in an illness whose exact causes are still largely unknown.

In glaucoma, antioxidant availability is impaired [57]. Since the first part of the nerve fiber is unmyelinated and particularly rich in mitochondria, a high production of ROS, not properly scavenged by antioxidants, could lead to an accumulation of oxidized forms of proteins, lipids, and DNA, both nuclear and mitochondrial [58,59]. In particular, the oxidation of cysteine residues on dynamin-related protein 1 (DRP1) can trigger mitochondrial dynamics, leading to an increase in mitochondrial fission that could result in excessive mitophagy followed by a drop in energy production. Consistently, Kim et al. showed in the DBA/2J mouse model of glaucoma that DRP1 inhibition, obtained by overexpressing DRP1 defective mutants, reduced both oxidation in RGCs and mitochondrial fission, suggesting a vicious cycle where exaggerated fission increased mitochondrial dysfunctionality, ROS production, and energy depletion [60]. On the other hand, defective mitophagy was found in experimental models of hypertensive glaucoma. Inducing chronic hypertensive glaucoma in rats by means of translimbal laser photocoagulation, Dai et al. showed that Parkin overexpression ameliorated optineurin expression, mitophagic flux, mitochondrial health, and RGCs survival in vivo, indicating that dysfunctional mitophagy could be involved in mediating hypertensive RGC damage [61].

Neuroinflammation plays a role in glaucoma. In glaucomatous human eyes, cells release several proinflammatory cytokines in the humor aqueous, including TNF-α [62]. An important role in glaucomatous diseases is attributed to the NOD, LRR, and NLRP3 inflammasome, which is formed by the assembly of several subunits, like cytosolic pattern recognition receptors, caspase1, NLRP, and the adaptor protein apoptosis-associated speck-like protein (ASC). The increased synthesis of inflammasome components, followed by their assembly, is an important part of the activation mechanism. Once activated, the proteolytic subunit of the NLRP3 inflammasome can convert the precursors of IL-1β and IL-18 into the fully active forms to be secreted. The activation of the NLRP3 inflammasome and IL-1β release was observed in mice after experimental IOP increase or an intravitreal injection of ATP, in particular in RGCs, Müller cells, and astrocytes, while NLRP3 KO mice were found to be protected against cell damage or death, indicating that the inflammasome could mediate an important part of the damage induced by IOP [63]. An interplay exists between mitochondrial dysfunction and inflammasome activation, represented by abnormal ROS release. In several cells and animal models, the use of inhibitors of mitochondrial complex I and III induced mitochondrial dysfunction and abnormal ROS release, followed by inflammasome activation and cell damage or death. Consistently, the antioxidant treatment of RGCs resulted in a significant reduction in NLRP3 activation and cell death in transient IOP increase models [64], suggesting that mitochondrial dysfunction is upstream to neuroinflammation, although in glaucoma this is not confirmed.

### 3.2. Diabetic Retinopathy

DR is a complication of diabetes mellitus, of both types 1 and 2. Different studies report variable DR prevalence in the population, with a higher incidence in the Middle East, North Africa, and the Western Pacific populations [51], being a leading cause of loss of vision and blindness. Hyperglycemia is recognized to be the core factor in DR etiology, affecting at first the retinal microvasculature, with a compromission of the BRB, and with subsequent increased vasodilation and permeability. The degeneration of microvasculature associated with pericyte loss determines the presence of capillary occlusion, ischemia, and hypoxic microzones that stimulates VEGF production from RPE or glial cells, which in turn prompts neovascularization and vascular permeability through alterations of tight junction components. This allows to distinguish two major types of DR, non-proliferative (NPDR) and proliferative DR (PDR). Initial NPDR is characterized by the presence of microaneurysms, hemorrhages, exudates, cotton wool spots, and intraretinal microvascular abnormalities. PDR presents further extensive ischemia, followed by neovascularization and the alteration of the retinal structure, that can lead to edema and retinal detachment, that increases the probability of visual loss. There is also evidence of an involvement of glial cells in the early stages of DR; in particular, hyperglycemic stress has been shown to activate microglial cells, that initiate an inflammatory response by secreting proinflammatory cytokines and VEGF [65]. Indeed, hyperglycemia deeply affects the different cell types present in the retina wall, triggering a neuroinflammatory response. It should be noted that the creation of an inflammatory environment in the retina has a causative role in DR and can be responsible for the finding that only half of the patients respond positively to anti-VEGF therapy, sustaining the need for integrative pharmacological approaches [66]. Accordingly, several cytokines and other inflammation markers, including ICAM-1, IL-1β, IL-6, IL-8, TNF-α, and MCP-1 have been found to increase in body fluids, like venous blood or aqueous and vitreous humor, in patients with DR [67,68]. Of note, the early pathogenesis of DR involves retinal neurodegeneration, so that recent evidence indicates it to be an independent event occurring before vascular damage could be observed [65]. Neurons and glia are both involved in the inflammatory response, which can recruit white blood cells from the blood to the retina, further prompting the formation of an inflammatory milieu, altering the BRB, and stimulating the cell death of photoreceptors and RGCs.

One accredited hypothesis calls into question glucose, present at high concentrations in the vessels and surrounding tissues. Glucose is an aldose sugar, able to react with proteins and lipids, giving rise to Schiff bases and other products, that collectively form the advanced glycation end-products (AGEs) [69]. It should be noted that AGEs, that can occur in all retinal cell types, not only directly damage almost all cellular components, but in addition are recognized by specific receptors, the most studied of which is the advanced glycation end-product receptor (RAGE). RAGE is a multiligand transmembrane receptor that, once activated, can upregulate NF-kB and its target genes, including inflammasome proteins, and a wide series of cytokines. In type-2 diabetes patients, without renal dysfunctions, the serum levels of AGEs were found to correlate with DR severity [70]. In particular, a role for the AGE-RAGE axis in the production of interleukins, VEGF, and ROS has been shown in in vitro cultured human RPE cells [71].

Extensive glycation, ROS production, and inflammation are responsible for initial RGC deterioration, which is further worsened by autophagic alterations [72]. Autophagy stimulation, triggered by ischemia/reperfusion, was found to be beneficial for RGC survival, and this was confirmed by the rapamycin- or starvation-stimulated upregulation of autophagy, which further improved RGC viability [73]. Coherently, the high-glucose treatment of isolated retinas leads to enhanced mTOR activation, which decreases autophagic flux. The neuroprotectant octreotide was effective in protecting against high-glucose-induced cell death through the inhibition of mTOR and the stimulation of autophagy [74]. In an immortalized cell line of RPE, N-acetyl cysteine treatment added before high glucose induction, reduced mitophagy markers to normal levels, suggesting the specific activation of mitophagy as part of the cell response mechanism to hyperglycemia [75].

While autophagy, as a whole, seems to play an overall protective role, mitophagy in DR appears to be a double-edged sword. In fact, it has been reported, in retinas obtained from hyperglycemic rats and in in vitro cultures in the presence of high glucose, that mitophagy increased in order to warrant the quality control of damaged mitochondria, but without being followed by an increase in mitochondrial biogenesis. The uncoupling of these two complementary parts of mitochondrial dynamic unbalanced mitochondrial turnover, leading to a dangerous depletion of the mitochondrial pool, suggesting the convenience of a strategy of mitophagy inhibition [76]. Interestingly, in in vitro, ex vivo, and in vivo models of multiple sclerosis, Patergnani et al. showed that the alteration of the mTOR/ULK1 pathway could induce abnormal glucose metabolism, mitochondrial dysfunctions, and uncontrolled mitophagy and autophagy. In the study, a strategy of autophagy inhibition with FDA-approved drugs not only greatly improved the ability of oligodendrocytes to restore axon myelination, but also warranted a significant recovery in in vivo behavioral tests [77]. In a similar way, Ealker et al., in another report, showed an overall convenience of autophagy inhibition to rescue motor neurons from death after cervical spinal cord injury [78]. Moreover, Du et al. [79] showed that in cell cultures challenged with high glucose, the use of autophagy inhibitors not only ameliorated cell viability but also reduced cell migration and tube formation, suggesting that autophagy could increase neovascularization, a dangerous side effect of DR. In a similar way, in a model of choroid-retinal endothelial cells, Piano et al. showed that the expression of PI3K, Akt, and mTOR was associated with autophagy inhibition and the diminution of high-glucose-stimulated vessel formation [80]. The link between high glucose, mTOR downregulation, and autophagy increase was also observed in an animal model where diabetes was induced using streptozotocin. The report showed that the inhibition of autophagy and the reduction of neuronal cell death could be achieved through glycemic control as well as specific mTOR activation by MHY1485 [81].

## 4. Therapeutic Applications of Extracellular Vesicles and Secretome Modulation from Mesenchymal Stem Cells in Retinopathies

Many experimental and clinical studies on retinopathies and, in particular, retinitis pigmentosa report positive evidence of the application of mesenchymal stem cells [82,83]. The added value of this strategy is created by the easy stem cell availability and, in general, by the fact that it does not induce serious immune responses [84,85,86].

**Figure 2:**
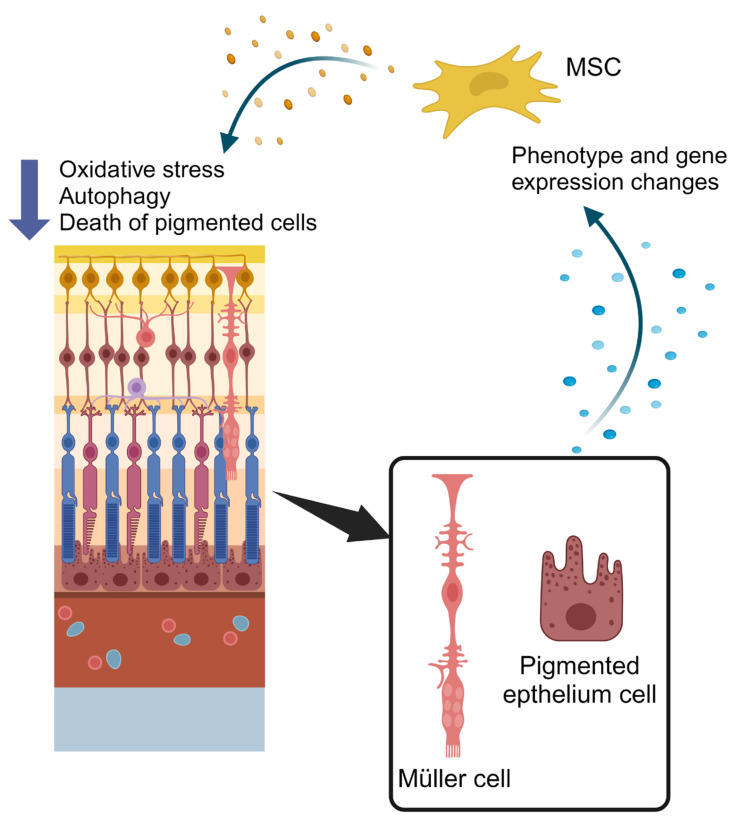
Schematic representation of secretome released by MSC and retinal cells. The crosstalk between MSC and Müller cells and pigmented epithelium cells allows the maintenance of the physiological morphology of the retina. Created with BioRender.com.

Studies underline that such therapies bring benefits to the surrounding tissue by secreting growth factors and extracellular vesicles to support tissue integrity, mainly because of their trophic and paracrine effects [82,87]. Notably, clinical research is also focusing on intraocular applications of mesenchymal stem cells, including subretinal, intravitreal, and suprachoroidal space, to prevent adverse effects such as the detachment of the retina and the epiretinal membrane [88]. In particular, several studies are aimed at assessing the role of a specific category of extracellular vesicles released by cells, called exosomes [89,90]. Exosomes represent an intercellular communication system, and are secreted in high quantities by mesenchymal cells, thus contributing to the therapeutic effect. In particular, the advantage of exosomes is that they do not contain cells, and this is a benefit at the immunological level with respect to cellular treatments. Several research studies in this field use Wharton’s jelly as a source of mesenchymal stem cells (MSCs), which is the mesenchymal tissue of the umbilical cord. This jelly represents a good source of stem cells with high differentiation and regenerative capacity, constant doubling time, high proliferation rate, and low immunogenicity. In addition, Wharton’s jelly can be easily obtained with non-invasive procedures and minimizes ethical problems [91,92]. MSC exosomes have a special biochemical composition since they also contain microRNA received from the secreting cell. Therefore, MSC exosomes can influence surrounding cells, modulating some pathways in the receiving cells. In particular, they are able to activate differentiation, stimulate proliferation, regulate the regeneration process, and, in general, increase cell survival (Figure 2) [93,94,95]. MSC-derived exosomes have already been used in experimental research to regulate RNA levels in certain types of cancer, including breast cancer and hepatocellular carcinoma [96,97]. Of considerable interest are the applications in diseases of the central nervous system, difficult to treat because of the anatomy of this body region that naturally limits the surgical treatment options. Exosomes allow researchers to overcome the blood–brain barrier and carry the molecule of interest to the target cells. To date, in fact, there is a great deal of information about exosomes used as transport systems for cytokine, growth factors, molecules of adhesion, nucleic acids, and chemotherapeutic agents. Exosomes might also be used in the field of gene therapy, as they can act as vehicles of therapeutic compounds like mRNA or siRNA. Attention is now focused on the use of exosomes as “therapeutic tools”, as recent studies have analyzed the proteomic and transcriptomic profiles of these vesicles and demonstrated that they express molecules on their surface: some of these are found in all exosomes (LAMP2B and CD63) [98,99], while others are fabric-specific. This can be exploited specifically to deliver exosomes loaded with selective drugs to certain target cells [100]. Drug delivery using exosomes can provide unique benefits compared to other systems, including limited immunogenicity and increased stability in the blood. Exosome membranes can also be modified to show epitopes that can bind to specific ligands present on selected cell types and improve their delivery to these cells.

By exploiting these peculiar features of exosomes, they represent an efficient system as vectors to accurately convey drugs to targets.

The morphofunctional integrity of the retina is subject to various levels of regulation, and a crucial role is played by the secretome of retinal cytotypes, especially RPE and Müller glial cells. It is also hypothesized that the secretome has the potential to affect the onset and progression of retinal diseases after an alteration of the secretory capacity of the cells [101].

Experimental data indicate that there is a crosstalk between the secretomes of mesenchymal stem cells and retinal cells [102]. Indeed, MSC secretome can modulate retinal cells, reducing oxidative stress, autophagy, and the apoptosis of pigmented cells. On the other hand, the secretome of retinal cells can affect mesenchymal stem cells by inducing changes in their phenotype and gene expression. Moreover, the comparison of the immunomodulating secretome produced by stimulated MSCs (st MSCs) with that of the unstimulated MSCs (Unst MSCs) without immunosuppressive effects, allowed researchers to identify their potential therapeutic properties. The results showed that stimulation with pro-inflammatory cytokines (IL1, IL6, and TNF) induced a significant change in the whole secretion of MSCs. In particular, most proteins, released exclusively from MSC activated with pro-inflammatory cytokines, are involved in the regulation of angiogenesis. These factors include metalloproteinase 1 tissue inhibitor (TIMP-1), a specific glycoprotein involved in the endogenous suppression of metalloproteinase [102]. By secreting TIMP-1, in fact, MSCs block the formation of new blood vessels in the draining lymph node, essential for the recruitment of circulating leukocytes to the inflamed tissue. Therefore, this event is responsible for the local suppression of the immune response [103].

In this regard, mesenchymal stem cells could be used as “biofabrics” of the secretome, to be used as mixtures that could have positive effects on retinal diseases [104].

## 5. Non-Coding RNAs as Innovative Diagnostic Tools and Therapeutic Molecules for Diabetic Retinopathy

### 5.1. Non-Coding RNAs

Cells produce a variety of RNAs, indispensable in the cellular processes that allow the cells to transform their genetic information into biomolecules that determine cell survival, adaptability, organization, and function.

However, other than the classical RNAs that take part in the transcription and translation processes (mRNA, rRNA, and tRNA), researchers have identified other types: these are classified as of non-coding RNAs (ncRNAs), that is, RNAs displaying a regulatory instead of protein-coding function. ncRNAs have different names and roles in cellular mechanisms, depending on their length, structure, and compartmentalization; the best known and described are micro (miRNA), long non-coding (lncRNA), and circular (circRNA) RNAs [105,106]. All of these are generated by RNA polymerase II, that can read both sense and anti-sense DNA sequences that code for ncRNAs. pre-miRNAs are subjected to post-transcriptional modifications, usually aimed at cutting bp to generate RNAs with ~20 nucleotides. They serve as regulators of mRNA expression: many miRNA binding sites have been identified in the 3′-UTR region of mRNAs; often, a single mRNA contains target sites for multiple miRNAs, but at the same time a single miRNA can bind multiple mRNAs. This generates a complex mRNA–miRNA network that revolutionized the model of the post-transcriptional regulation of gene expression [105].

lncRNAs are longer (more than 200 nucleotides) and transcribed from intergenic or intronic regions of genes, or even from the antisense filament of these. They mainly act as scaffolds or decoys for protein and regulatory molecules, such as the very same miRNAs, modulating their abundance and consequently their effect on gene expression. circRNAs are another type of longer ncRNA, since their sequences can range from 1 to 5 exons, including up to 4 introns. This characteristic grants them the same functions as lncRNAs, but they have also been shown to be used as templates for protein synthesis, in place of mRNAs [105,106,107]. However, this mechanism needs further study to be fully appreciated and to understand its biological relevance.

Despite being studied for several decades, it is quite a recent discovery that these ncRNAs also exist in a cell-free form (cfRNAs), dispersed in various body fluids, and even more recent is their application for diagnostic and therapeutic purposes [105]. The role of these cfRNAs is still debated, although many seem to agree that this form represents a way of intercellular communication, that can be paracrine or even endocrine, paving the way for a more complex and inter-tissue regulation of gene expression [105,106].

### 5.2. ncRNAs Involvement in Diabetic Retinopathy

ncRNAs have been extensively studied for their role in shaping the functions of the RPE in hyperglycemic conditions, as they occur in diabetic patients and are involved in the development of DR. The RPE has the function of supporting photoreceptor cells and maintaining the homeostasis of the retinal tissue with continuous exchanges with the vasculature. ncRNAs of every type have been implicated in modulating the RPE structure and functions, in particular those involving interactions with blood vessels [107,108]. Endothelial cells are another cell type severely affected during retinopathy development: in fact, during these processes, the normal vasculature is damaged by pro-inflammatory stimuli, and abnormal vasculature formation is promoted by biomolecules released by other cell types, the most prominent being VEGF. Human retinal vascular endothelial cells (HRVECs) that sense damage signals will activate, proliferate, and migrate abnormally, altering the function of retinal blood vessels. Other than modulating intracellular signaling pathways that regulate VEGF release from non-endothelial cells, ncRNAs also have a role in determining the survival and proliferation of endothelial cells themselves [107]. Inflammation has a prominent role in retinopathies, including DR, and it is well established that ncRNAs are involved in the regulation of this mechanism. Some of these ncRNAs have been studied for their role in the NF-kB pathway, one of the most prominent pathways activated during inflammation [108,109,110,111,112]. In some cases, the in vitro results were supported by RNA analysis on the serum of diabetic patients, as in a study that revealed the positive correlation between miR-146a-5p downregulation and DR progression, showing its relevance in a clinical setting [112]. Other miRNAs were associated with the downregulation of pro-inflammatory and pro-angiogenic signals (e.g., TNF-α and IL-1) [113,114], while some circRNAs seemed to be involved in a deleterious, proinflammatory response [115,116].

Inflammation is usually accompanied by cell death, that can help to sustain the inflammatory response and participate in tissue damage. ncRNAs have a variety of interactions with several pathways involved in cell survival and apoptosis. Different ncRNAs have proapoptotic effects on RPE cells [117,118,119,120], causing cell dysfunction, apoptosis, and the reduction of the thickness of the retinal barrier; others are antiapoptotic, but they are found downregulated in hyperglycemic conditions [108,121]. Apoptosis may also involve endothelial cells, compromising the healthy vasculature and promoting pathogenic neovascularization; in this context, an important protein for endothelial cell survival seems to be SIRT1, a histone deacetylase associated with antiapoptotic signaling, thus downregulated by different ncRNAs [116,122,123]. Other downregulated pathways involve TGFB2 and FGF2 [124].

HRVEC dysfunction can also be a consequence of the alteration of the crosstalk with pericytes, that can be influenced by ncRNAs. Some ncRNAs prevent pericyte degeneration, and consequently vascular dysfunction, like MEG3, that showed important anti-inflammatory and antiapoptotic activity [125,126]. circRNAs seem to have protective effects on pericytes [127], with circEhmt1 also being transferred via the exosome to endothelial cells, inhibiting inflammatory and apoptotic processes [105,106]

In DR, an imbalance in growth factor levels is also observed: sustained VEGF release is dangerous for the vasculature, causing damage and the leakage of the BRB in the early stages, and promoting neovascularization in the proliferative stage. Recent studies have shown that an intricate network of ncRNAs may contribute to or counter this abnormal activation of the VEGF pathway, both in secreting and responding cells. In regard to its pathogenic role, another study showed a significant correlation between the decrease in the expression level of the aforementioned lncRNA MEG3 in the serum of DR patients and the increase in VEGF serum levels [128]. In the same study, a similar correlation was observed for TGF-β serum levels, pointing to a broad involvement of MEG3 in DR pathogenesis, and its potential role as a therapeutic target. Other than this particular case, researchers observed a great number of ncRNAs able to downregulate VEGF, control the abnormal proliferation of endothelial cells, and block the progression of PDR in endothelial cells exposed to HG [107,108,129,130]. Also, the histone deacetylase SIRT1 positively regulates the expression of miR-20a, inducing HIF1α to downregulate VEGF activity and prevent the abnormal proliferation of endothelial cells when exposed to HG in vitro [131]. However, we also need to consider a significant number of ncRNAs with the opposite activity, that regulate VEGF pathway to make endothelial cells proliferate, thus facilitating the formation of new blood vessels and aggravating the PDR process [107,108,132]. lncRNAs and circRNAs majorly favor VEGF signaling, since they act as sponges to reduce the levels of anti-VEGF miRNAs [120,133,134,135,136,137,138]. On the other hand, some lncRNAs have protective effects, like TPTEP1, that reduces VEGFA expression and therefore neovascularization [139]. Another target heavily modulated by ncRNAs is the PI3K/Akt pathway [140,141,142,143,144], responsible for cell proliferation and tube formation potential.

Other than directly targeting endothelial cell proliferation and neovascularization, some ncRNAs are responsible for altering the functions of the BRB. Researchers found that ncRNAs can also correlate with an increased vascular permeability [133,145]. Different studies have reported the regulation of the same pathway by a number of miRNAs: miR-411, miR-125b-5p, and miR-146a-5p all target ROBO4 to maintain vasculature and RPE functions, but they are downregulated in hypoxic and hyperglycemic conditions, and this suggests that they might be suitable targets to ameliorate RPE cell dysfunction [146,147].

### 5.3. ncRNAs as Predictive Biomarkers and Novel Therapeutics

Since ncRNAs can be released by cells into the general circulation, especially in the case of tissue damage or diseases, many have proposed to study their application as disease biomarkers, due to their high stability in the bloodstream and the ease of collecting blood samples. Despite all the studies highlighting the importance of ncRNAs in several diseases, this field seems to be lagging behind: considering that even for cancer diagnosis, there is still no approved protocol to evaluate ncRNA levels in liquid biopsies to assess disease stage/progression, no significant advancements have been made for retinopathies [105]. There are multiple reasons, some of which are related to the specific disease, but this discussion does not belong to the scope of this review. Instead, we desire to focus on the ncRNAs that have been indicated as potential biomarkers for retinopathies.

In a prospective study analyzing miRNA levels before disease onset in patients with type 1 diabetes (T1D), Zampetaki et al. found an independent association between miR-27b and miR-320a and the risk of developing DR, although they are not retina-specific and could indicate a systemic predisposition to pathological angiogenesis [108,148]. Another study showed not only that decreased levels of circulating miR-126 were associated with DR, but that it was also possible to distinguish between progressive and non-progressive DR [149]. miR-126 downregulates IRS-1 to inhibit the PI3K/Akt pathway and therefore the viability and invasion of endothelial cells [150]. miR-211 was found significantly elevated in DR T1D patients, that is consistent with its role in inhibiting SIRT1 and promoting endothelial cells apoptosis [151]. For type 2 diabetes (T2D) patients, researchers indicated a significant increase in circulating miR-93 as a biomarker for DR [152], associated with increased levels of VEGF given its role in the regulation of its expression [153]. Interestingly, a group observed that miR-122 expression increased during disease progression, with significative differences between healthy controls and T2D patients with no DR (NDR) and between NDR and NPDR patients; conversely, in PDR, an unexpected, marked decrease in miR-122 levels was reported. miR-122 is associated with an inhibition of proliferation and angiogenesis, so this finding is coherent with the effect of this miRNA on endothelial cells [154]. Similarly, a progressive increase in serum levels of miR-221 was found to be a better predictor of NPDR and PDR, even when compared to serum VEGF, in T2D subjects [155,156]. Mazzeo et al. performed a wide molecular and functional characterization of extracellular vesicles secreted from mesenchymal stem cells in DR conditions, identifying miR-150-5p, miR-21-3p, and miR-30b-5p as significantly increased when compared to the levels contained in EV from healthy subjects, therefore indicating these miRNAs as potential biomarkers [157].

As we have briefly mentioned, when talking about ncRNAs associated with retinopathy development, it is possible to modulate the levels of some of these molecules to yield a beneficial effect in DR. This can be achieved by either using antagonist molecules to inhibit pro-DR endogenous miRNAs or miRNA mimics to rescue the anti-DR pathways [108,158].

For example, miR-145-5p targets the FGF5 gene, whose downregulation promotes DR progression. Researchers reported that the miR-145-5p inhibitor was able to exert protective effects by restoring FGF5 expression, decreasing proinflammatory cytokines (TNF-α, IL-6), and improving cell viability and proliferation [159]. Another study showed that the miR-183 inhibitor was able to inhibit the PI3K/Akt/VEGF pathway in endothelial cells, suppressing proliferation and angiogenesis [132]. Also, some compounds of natural origin have been tested in attempts to address the elevated inflammation and apoptosis in RPE cells. Hawthorn polyphenol extract is able to reduce oxidative stress, apoptosis, and inflammation in RPE cells exposed to high glucose concentrations by inhibiting the miR-34a/SIRT1/p53 axis. In particular, it downregulates miR-34a, releasing SIRT1 that, as previously reported, prevents apoptosis [159]. Similarly, Astragalus polysaccharide targets miR-204, inhibiting its activity, thus releasing SIRT1 and preventing RPE cell apoptosis [160]. The same molecule has been shown to inhibit miR-195 and simultaneously alleviate oxidative stress and mitochondrial damage in RPE cells in HG conditions. miR-195 downregulates Bcl-2, promoting the intrinsic apoptotic pathway; thus, Astragalus polysaccharide is able to restore the activity of this key antiapoptotic molecule [161]. Similarly, melatonin acts on Müller cells, modulating their activation and the release of pro-inflammatory cytokine through the upregulation of the lncRNA MEG3; also, this lncRNA targets the miR-204/SIRT1 axis in in vitro models of DR, reducing miR-204 levels [162]. Resveratrol also acts on Müller cells, decreasing diabetes-induced apoptosis by upregulating miR-29b expression in a rat model of DR [163]. Chen et al. identified a miRNA downregulated in DR, miR-144-3p, and showed that its mimics were able to inhibit cell proliferation and VEGF activation in endothelial cells, through the inhibition of the FGF16 and MAPK pathways [164]. miR-384-3p mimics were studied after Xia et al. found that the levels of this miRNA were reduced in DR mice, while hexokinase 2 (HK2) expression was increased. Treatment with miR-384-3p mimics of DR mouse-derived RMECs demonstrated to be effective in reducing HK2, cell proliferation, and tube formation [165].

Despite this body of evidence, studies are still ongoing to find novel targets, develop combined ncRNA strategies, and optimize delivery for in vitro and in vivo trials.

## 6. The Gut–Retina Axis: A New Perspective in the Study of Retinal Dysfunction

The ocular eubiosis, intended as the correct balance of microbiological and microbiomic composition, physiologically presents a stable composition. Recent evidence has pointed out that the bacteria present on the eye surface can play a role in maintaining homeostasis by modulating the immune function and protecting from infections in some conditions [166,167].

### 6.1. Ocular Microbiota

Compared to other anatomical areas, the bacteria present in the eye area are much less numerous. This is due to the presence of powerful antimicrobial factors in the tear fluid and the constant mechanical action of the eyelids, which eliminates microbes. Despite this, many microorganisms survive on the eye surface [168,169]. Indeed, the ocular surface presents a microbial flora, formed by Gram+ and Gram− bacteria which colonize the ocular surface immediately after birth. The healthy ocular microbiota was analyzed using metagenomic analysis, and nine predominant taxonomic profiles emerged [170]. In particular, it has emerged that the most present bacteria are Staphylococcus, Bacillus, Corynebacterium, Pseudomonas, Kocuria, Aerococcus, and Chryseobacterium [171,172].

The composition of the eye microbiota can be affected by several factors: the use of contact lenses, cosmetics, drugs, infections or diseases affecting other organs, age, and climate (dry or wet) [173]. Physiologically, the eye surface has no protection from the environment and is more susceptible to microbial contamination than other areas of the body. During evolution, microbes colonized the eye surface and became commensal bacteria. Despite this interaction, the epithelial cells of the cornea and conjunctiva are not prone to develop inflammation or pathological states in healthy subjects. This might be explained by a specific immunological tolerance towards commensal bacteria. These bacteria are also responsible for the homeostasis and the prevention of colonization by pathogenic species. Indeed, the alteration of this equilibrium is linked to a number of inflammatory diseases of the eyes [174].

Studies in this field are still preliminary, but early developments suggest that alterations of the ocular microbiota could be the cause of pathologies such as dry eye, blepharitis, and others. If these data are confirmed, in the next few years, the characterization of the ocular microbiota could become an additional test to complete the diagnosis and to discover useful information to guide the therapy.

Dry eye, for example, is a common condition, caused by the inadequate lubrication of the eye by tears [175,176]. This condition causes inflammation and damage to the eye surface, without an obvious infection. In some cases, bacteria present on the eye produce lipases and toxins that modify the tear film, causing signs of irritation, as in the case of patients with dry eye. These subjects have higher levels of Bacteroidia and Bacteroidetes on the eye surface, and lower levels of Pseudomonas and Proteobacteria, compared to subjects with healthy eyes. Such variation of the ocular microbiota is a possible cause of dry eye [177]. In the same way, blepharitis, the inflammation of the eyelids, of a different pathogenic origin, seems to be associated with an increased amount of bacteria Corynebacterium and Enhydrobacter.

The ocular microbiota of diabetic patients are more relevant than those of healthy subjects. In patients with diabetes, the tear film may contain higher levels of glucose, and this facilitates infections by potential pathogenic bacteria. It has been shown that an increase in coagulase-negative Staphylococcus bacteria is associated with DR [178]. It is possible that the inflammatory cytokines released in diabetic patients also influence the eye microbiota, favoring the growth of pathogenic bacteria on the eye surface, that in turn promote and sustain the inflammatory response, that may play a role in DR onset and progression. Thus, studying the eye microbiota and how we can protect and sustain the commensal bacteria in diabetic patients might become a useful strategy to combine with other treatments in the prevention of DR development.

### 6.2. Gut–Eye Axis

Studies on the interaction between the ocular and intestinal microbiota have increased in recent years. Some researchers hypothesize the existence of a gut–eye “axis”: the alteration of the intestinal microbiota seems to be linked to some eye diseases, including uveitis, macular degeneration, and glaucoma [179,180,181,182]. Most microorganisms are located in the intestine, especially in the ascending colon: therefore, we can find bacteria, fungi, viruses, and helminths in constant symbiotic relationship with our immune system and with numerous functions, such as metabolic, protective, of immune education, and of vitamin production [183]. It should be emphasized that when the quantity of saprotrophic microorganisms grows and exceeds the limits, they can become pathogenic; but since this growth is often slow, it does not determine reactions of the immune system; instead, it becomes a silent infectious trigger for chronic and immune inflammatory diseases. However, transient microorganisms, acquired with food, air, and contact with the outside world, could pass through and compete with resident ones in certain conditions, becoming inflammatory triggers. The intestinal mucosa must be unobstructed and intact to avoid the passage of toxic substances and immunostimulants from the intestinal lumen to the bloodstream, in order to prevent the occurrence of immune-mediated diseases by the intestinal and oral microbiota [184].

The rationale of the action of the intestinal microbiota on organs distant from the gut, like the eyes, is in the production of immunomodulatory cytokines by intestinal immunocompetent cells (e.g., Peyer Plaques). The balances between Th1/Th2 and Th17/Treg, respectively, are regulated by cytokines produced by the immune cells themselves in a positive or negative way [185]. The general principle of this axis, therefore, is based on evidence that the dysbiosis of the intestinal microbiota induces an increased production of inflammatory cytokines and other systemic inflammatory mediators that can act at a distance on some target structures. The presence of inflammatory substances alters the functionality of the lymphoid tissue associated with the mucous membranes, also damaging the conjunctival mucosa, located in the eye [186].

Numerous scientific studies underline that the microbiota and the intestine must be in balance in order to have a general homeostasis of the organism. In fact, alterations of the crosstalk between host and intestinal microbiota can lead to a low-grade inflammation, the starting point of systemic diseases and a contributing factor to numerous pathological conditions [187,188,189,190].

The main mechanisms proposed to explain the gut–eye axis are various: the activation of dendritic cells and migration to the lymph node, the activation of Th1/Th17 cells in the intestine and self-reactive B cells with migration into the eye surface/lacrimal gland, and the antigenic mimicry of bacterial products resulting in autoimmunity [191].

### 6.3. Potential Effectiveness of Probiotics and Prebiotics

In the past decade, many studies have shown the effectiveness of probiotics and prebiotics. Recently, some research studies have been published about the modulation of the microbiota in ophthalmology and the treatment of eye diseases. Chisari et al. carried out a pilot study to establish the effect of probiotic administration on the eye surface, with the supplementation of Bifidobacterium lactis and Bifidobacterium bifidum [192]. At 30 days, in the treated group, they observed a statistically significant improvement in the Shirmer test and the break-up time, together with a reduced colonization of the ocular surface by *S. aureus*.

Moreover, a further interesting result was obtained with the topical use of different types of probiotics based on *Lactobacillus acidophilus*. Lactobacilli are non-pathogenic, Gram + microorganisms, and the main components of commensal microbial flora that, in several clinical studies, have shown efficacy in the adjuvant treatment of various allergic symptoms, also in ophthalmology. A pilot study, carried out on patients of pediatric age with keratoconjunctivitis, demonstrated the effectiveness of a galenic preparation for ophthalmic use based on thermally inactivated *Lactobacillus acidophilus*, that reduced signs and symptoms of the disease. The results obtained demonstrated a reduction in the parameters examined and a downregulation of the adhesion molecule ICAM1 and of the pathogen recognizing receptor TLR4 in the conjunctival epithelium, in order to counterbalance the Th2 mediate immune response typical of allergic diseases [193].

An additional study on LP has demonstrated the anti-inflammatory effect on a model in vitro, finalized to characterize treatments for patients refractory to the conventional therapies [194].

Future studies will be aimed at identifying “healthy” eye microbiota, their normal ranges, and therapies to correct the microbiotic imbalance.

## 7. Human Retinal Organoid: Is It a Useful New Tool for Preclinical Studies?

### 7.1. Retinal Organoids

Organoids represent one of the most advanced tools to generate new models that more closely mimic physiological and pathological conditions. For this reason, they are platforms to study pathogenic mechanisms and identify effective therapeutic targets, also in the context of personalized medicine [195,196]. Organoids are 3D multicellular structures that can be established from embryonic or adult stem cells, that under precise settings self-organize and differentiate in 3D cell masses with characteristics very similar to, and sometimes histologically indistinguishable from, those of in vivo organs [196,197]. Because of their characteristics, organoids can be defined as cultures that exhibit multi-cellularity and functionality, show spatial architecture like in vivo organs, and preserve stem cells or progenitor pools [198]. Since 2009, when for the first time a small intestinal organoid was established in vitro [199], several 3D cultures were obtained from different organs, such as the kidney [200,201], liver [202,203], brain [204,205], and retina [206,207], by changing the combination of growth factors and cell isolation procedures. The organoids can be classified into three types, based on different characteristics, such as types of cells and how they interact with each other: they are classified as epithelial, multi-tissue, and multiorgan [208]. Epithelial and multi-tissue organoids are derived, respectively, from cells of a single or at least two embryonic leaflets; in the latter case, they can mimic the organization of the original organs that are normally formed by different tissues. Multi-organ organoids are formed by cells from two or more organs and simulate the physiological interaction between them [209,210,211,212]. During embryogenesis, the optic vesicle originates from neuroectoderm and, as a result of the invagination of the front of the vesicle, a double layered structure is formed. This structure, named optic cup, presents two adjacent epithelia: the inner one is the neural retina, and the outer one is the retinal pigment epithelium [213]. The retina is composed of five types of neuronal cells (photoreceptor, horizontal, amacrine, bipolar, and ganglion cells) that are distributed to form a complex laminated structure. Since the 1960s, scientists have proven the capacity of dissociated chicken retina to reaggregate and self-organize in vitro [214], to form a spheroid [215], and to organize into a laminated structure when treated with Wnt2b [216]. A great step forward was achieved when Sasai’s group obtained an optic cup starting from mouse and human stem cells [217,218] which then differentiated in 3D retinal organoids (ROs). The differentiation was induced by treating the cells with various exogenous factors and, so far, many research groups have adapted Sasai’s protocol to obtain ROs with functional cone and rod photoreceptors [219,220] and to establish a cone-rich model able to mimic the human macula [221]. A critical point in the use of ROs as study models for retina diseases is the inability to correctly incorporate a representative retinal pigment epithelium layer, whose interaction with photoreceptors is essential for the functioning of the neuroretina [222,223], a problem that was not encountered in models of retinal explants containing neural retina and RPE cells [224]. Different studies have shown that when human induced pluripotent stem cells (hiPSCs) were treated with RPE-derived factors, or cocultured along with RPE cells, it was possible to observe an improvement in the maturation of organoids and in the differentiation of photoreceptors [224,225]. Recently, Usui-Ouchi and colleagues [226] have generated mature ROs with integrated macrophage precursor cells, thus producing a model implemented with microglial cells that may be of great importance in the study of retinal pathologies involving retinal microglia.

### 7.2. Organoids as Models for Retinal Degenerative Diseases

The human retina has no regenerative capacity, and retinal degenerative diseases (RDs) are the main causes of vision loss and blindness worldwide [227,228]. In this context, the discovery and introduction of new therapeutic approaches is of fundamental importance. A central issue in the study of retinal pathology is the absence of proper models that can mimic the structural complexity of the retina. Indeed, in vitro 2D cell cultures are not adequate to be representative of the multilayer structure of the retina, while the interspecies differences often make the use of animal models inappropriate to simulate human RD phenotypes; for instance, the mouse retina does not present a fundamental structure such as the macula [229].

The lack of appropriate models can be overcome using 3D ROs, derived from embryonic stem cells or induced pluripotent stem cells (iPSCs), that recapitulate the retinal structure and the cell–cell interactions, thus representing a good model for the study of the pathophysiology of the retina [230,231]. In several investigations, ROs proved to be useful as models of inherited and non-inherited retinal diseases and for the study of genome-editing technologies.

Patient-derived iPSC organoids were successfully used for the study of retinitis pigmentosa (RP), a congenital inherited retinal dystrophy characterized by many disease-causing genetic mutations [232]. With the development and implementation of production techniques of iPSC-derived ROs, it was possible to evaluate the impact of mutations in different genes involved in the development and progression of this pathology [233,234,235]. The more common causes of RP are the mutations of the X-linked retinitis pigmentosa GTPase regulator (RPGR) gene. In a study of Deng et al. [236], organoids derived from three different patients with mutations in this gene were generated and used as models for the study of the efficacy of the gene target therapy to counteract the structural defects typical of the pathology. The authors demonstrated that the correction of RPGR gene mutation mediated by CRISPR-Cas9 preserved the photoreceptors’ structure and electrophysiological properties, counteracted ciliopathy, and restored gene expression. In recent research, organoids were used to evaluate the impact of several genes on RP development. In addition to RPGR [237], other genes have been studied such as pre-mRNA processing factor 31 homolog (PRPF31) [238,239] and Crumbs Cell Polarity Complex Component 1 (CRB1) [240,241]: in all these studies, CRISP-Cas9 gene editing was used to restore gene functionality. Other authors [242] have shown that the use of Adeno-associated viral vectors was also able to restore the phenotype of organoids derived from CRB1-mutated patients.

Hirami et al. have studied the effects of transplanting an allogenic iPSC-derived RO sheet in two patients affected by RP [243]. In the study, they demonstrated that the graft was stable for two years, without causing serious adverse effects. Furthermore, in the transplanted eye, the pathology progressed more slowly than the opposite eye, used as control, suggesting that the transplantation of ROs should be investigated as a new therapeutic approach.

Organoids were also used for the study of other inherited pathologies such as Stargardt’s disease [244], Leber congenital amaurosis, and Joubert syndrome-related disorders [245].

Among the non-inherited retinal diseases, many authors have used organoids for the study of retinoblastoma (Rb). Rb is a childhood tumor which has an incidence of 17% among all pediatric cancers [246]. The use of ROs for the study of Rb was of great importance because it allows the differences between animal models and the human retina to be overcome. Indeed, in animal models, the origin of the tumor is attributed to Müller glial cells, amacrine, or horizontal cells [247,248], while in the human retina, it seems involve cone precursors [249], as also supported by studies conducted with the use of ROs derived from hESCs mutated on the RB1 gene [250]. recent years, several studies have been conducted in ROs derived from hESCs or iPSCs harboring an RB1 mutation or inactivation, allowing researchers to elucidate the underlying pathogenetic mechanisms and the transcriptional events involved in Rb [246,251,252,253]. Recently, Gabriel et al. [254] demonstrated that organoids obtained from iPSCs were capable of forming bilaterally symmetrical optic vesicles. The optic-vesicle-containing brain (OVB) organoids consisted of different cell types belonging to developing optic vesicles such as retinal progenitor cells and pigment epithelial cells, suggesting the possibility to use OVB organoids as models for retinal diseases caused by neurodevelopmental disorders.
